# Drug use and risk behaviours among injecting drug users: a comparison between sex workers and non-sex workers in Sydney, Australia

**DOI:** 10.1186/1477-7517-2-7

**Published:** 2005-06-06

**Authors:** Amanda Roxburgh, Louisa Degenhardt, Courtney Breen

**Affiliations:** 1 National Drug and Alcohol Research Centre University of New South Wales, Sydney, NSW 2052, Australia

**Keywords:** sex workers/prostitutes, patterns of drug use, risk taking behaviour, injecting drug users

## Abstract

**Background:**

This paper examines the differences in demographics, drug use patterns and self reported risk behaviours between regular injecting drug users (IDU) who report engaging in sex work for money or drugs and regular injecting drug users who do not.

**Methods:**

Cross sectional data collected from regular IDU interviewed as part of the New South Wales (NSW) Illicit Drug Reporting System (IDRS) in 2003 were analysed.

**Results:**

IDU who reported engaging in sex work were more likely to be female, and identify as being of Aboriginal and/or Torres Strait Islander descent. They initiated injecting drug use at a significantly younger age and were more likely to report injection related problems than IDU who had not engaged in sex work. There were no differences in the drug classes used, but findings suggested that the sex workers tended to be more frequent users of crystalline methamphetamine (ice) and benzodiazepines.

**Conclusion:**

The similarities between these groups were more striking than the differences. Further research, examining a larger sample is needed to clarify whether injecting drug users who are sex workers have heavier use patterns.

## Background

The last two decades have seen an increasing interest in the study of sex workers as a marginalised group at increased risk for poorer mental and physical health outcomes, inequitable access to housing and the problematic use of illicit drugs [1]. Previous research has documented the risks of blood borne virus (BBV) transmission and sexually transmitted infections among sex workers due to unprotected sex with clients [2], the relatively high rates of HIV among sex workers in some countries, and the potential risks posed to the broader community via BBV transmission through clients to the general population [3]. It should be noted that HIV prevalence among sex workers differs in Asian countries compared to North America and Europe. In the latter countries research has shown that HIV prevalence is no different among IDU and sex workers who are IDU [4], and that HIV is more prevalent among sex workers who are IDU compared to sex workers who are not IDU [5], indicative that it is injecting drug use that puts these groups at risk of HIV. In countries such as Africa, HIV infection is largely associated with heterosexual activities [6].

The literature suggests that sex workers are disadvantaged across a number of domains. One study that examined mental health status among a group of Puerto Rican sex workers [7] found that the overwhelming majority (91%) reported a high rate of depressive symptoms (measured using the Centre for Epidemiologic Studies Depression Scale). The authors also found that street sex workers reached significantly higher levels of depressive symptoms (86%) than brothel workers (45%). Approximately half of the sample (47%) reported injecting drug use, and a significantly higher proportion of those injecting drugs (90%) reported high levels of depressive symptoms compared to non injecting drug users (52%).

Another study comparing street sex workers and non street sex workers in Sydney, Australia [8] found that street sex workers were; predominantly female, significantly more likely to identify as being of Aboriginal and/or Torres Strait Islander descent (20%), and to be currently injecting drugs (77%) than those working `indoors' (7%). Street sex workers also had high rates of Hepatitis C (71%), possibly indicative of their injecting drug use. Interestingly, a significantly higher proportion of `indoor' sex workers (48%) reported alcohol use than street sex workers (29%). Approximately one quarter of the street sex workers had no permanent accommodation, and a similar proportion (27%) reported no supportive relationships.

In contrast to these findings, a study comparing sex workers and non sex workers in New Zealand [9] found no differences between the groups across domains such as access to accommodation, level of social support, and mental and physical health. However a significantly higher proportion of sex workers (76%) reported tobacco use than non sex workers (29%) and sex workers also reported higher consumption of alcohol (58% reported drinking more than 5 standard drinks per occasion of use compared to 23% of non sex workers). The absence of differences between sex workers and non sex workers in this study may be attributed to the fact that only 2 of the sex workers sampled were street workers. Previous studies suggest that street sex workers are a more marginalised group than non street sex workers, and if the sample contained a greater number of street sex workers, the authors may have found more significant differences on a range of variables.

These patterns are also evident among male sex workers. An Australian study sampled male sex workers in three cities (Melbourne, Sydney and Brisbane) to document their characteristics [10]. When street sex workers were compared with non street sex workers, they were less educated, more likely to report financial problems, less likely to be tested for blood borne viruses and sexually transmitted infections, and were higher drug users than non street sex workers.

These differences are also evident in the U.K, with studies showing a higher prevalence of injecting drug use and more problematic drug use, among street sex workers compared to non street sex workers. There is also evidence in the U.K. literature of women moving to street based sex work from indoor markets due to problematic drug use [11].

Research reviewed (e.g. [7]) indicates that drug use is an important predictor for poorer outcomes for sex workers, which has generated an interest in the role of drug use, and drug use patterns among this group. An ethnographic study of women in New York who engaged in sex work [12] found that drug use played a substantial role in the way these women conducted their sex work. Crack cocaine had a particularly deleterious effect on sex workers as it was thought to lead to lowering of the price of sex work exchanges, engendering a more hostile environment among sex workers and more violent exchanges with clients, and an increased potential for high risk sexual encounters. Many of the women Maher interviewed also used crack in order to facilitate their engagement in sex work.

One study of a group of cocaine `dependent' sex workers in the United States [13] found that two thirds of the sex workers came from ethnic minority groups, two thirds had completed less than 12 years of education, and a fifth were homeless. Another U.S. study, investigating "crack" cocaine smoking sex workers [14] mirrored these results, as did an Australian study [8] in which 20% of street sex workers identified as Aboriginal and/or Torres Strait Islanders and a fifth had no permanent accommodation.

The risks faced by sex workers are further compounded by drug use, with studies documenting associations between sex workers' drug use and the poorer safety outcome of the sex encounter (e.g. [15]), and risk of BBV transmission due to injecting drug use and sharing of needles [2]. Sex workers are a group characterised by high levels of drug dependence and those who inject drugs may be at greater risk on a multitude of factors than sex workers who do not. A study of 51 female sex workers in London who were current drug users [2] found that the majority of women using heroin (88%) were daily users, and many reported high levels of dependence in accordance with the Severity of Dependence Scale (SDS). The majority of IDU sex workers (75%) had used injecting equipment after someone else. However, the sharing of injecting equipment was related to severity of dependence on heroin rather than sex worker status *per se*. This finding suggests that dependent drug use may be a key factor for engaging in risk behaviours rather than sex work.

Comparative research has been conducted in Australia examining drug use among sex workers and various other groups, including women from community health services [16] and women from general population surveys [17]. Findings suggest that female sex workers have higher rates of illicit drug use [16], heavier use of alcohol and tobacco [9,16,17] and higher rates of sharing injecting equipment [17] compared to women from the general community. While these findings provide some insight into drug use patterns among each group, they have tended to sample non sex workers from populations that are likely to be quite different across a number of domains than sex workers, therefore limiting the validity of comparisons and conclusions about the risks that involvement in sex work may carry.

One U.S. study has examined similar groups. Logan, Leukefeld and Farabee [18] investigated the differences between female crack users according to whether or not they engaged in sex work, and found that both groups were just as likely to be African American, to be unemployed, to have similar educational backgrounds, and similar drug use patterns. However, women engaging in sex work were likely to have less access to accommodation, more frequent contact with the criminal justice system, earlier initiation of alcohol and cocaine use and higher rates of injecting drug use than non sex workers. In summary, while these groups were similar in some respects, there were also important differences, indicating that among an already marginalised group (i.e. crack users) sex workers are more likely to be even more marginalised than their non sex worker counterparts.

Investigating differences between sex workers and non sex workers among injecting drug users (IDU) may provide new insight into whether sex work status is likely to increase the risks among an already marginalised group. To the best of the authors' knowledge there has been no research conducted in Australia among IDU to determine whether there are differences between sex workers and non sex workers with regard to drug use patterns and risk behaviours. Previous research in Australia has focused on sex workers and their drug use patterns without comparison data. Australian findings are likely to differ from studies conducted in America, as there is little, if any, crack use in this country [19], and heroin is the most commonly injected drug among sentinel groups of regular IDU, particularly in Sydney [20,21].

The current study aims to examine whether regular injecting drug users who engage in sex work are at greater risk for adverse outcomes (such as homelessness and poor mental health), are more likely to engage in risky behaviours (needle sharing, criminal activity), and have different drug use patterns than injecting drug users who do not engage in sex work. Data are drawn from the Illicit Drug Reporting System (IDRS), in which sentinel groups of regular IDU are sampled annually.

### Aims

1. to document the proportion among a sentinel group of regular IDU who report engaging in sex work for money and/or drugs;

2. to compare the demographics of this group with regular IDU who do not report sex work;

3. to examine and compare the drug use patterns of these groups;

4. to consider and compare self-reported risk behaviours in these groups.

## Method

This paper used cross-sectional survey data collected in 2003 on a sentinel population of regular IDU regarding their drug use history, patterns of use, risk taking behaviours and drug-related harms. Data were from the NSW Illicit Drug Reporting System (IDRS). The IDRS is conducted annually in June using the same methodology, and provides sensitive data on trends and changes in drug use over time [22].

Participants were recruited through Needle and Syringe Programs (NSPs) in Sydney, NSW. NSP sites were chosen due to their proximity to street based illicit drug markets, as these markets are likely to attract regular IDU. Interviewers were positioned in the waiting area of the NSP, and clients were asked if they were interested in participating in a confidential survey being conducted by the University of New South Wales. Although some clients declined to be interviewed, refusal to participate did not present as a major issue. Participants received reimbursement of $30 for travelling expenses. Eligibility criteria for entry into the study were: (i) at least monthly injection in the six months preceding the interview; and (ii) residence in Sydney for twelve months preceding the interview, with no significant periods of incarceration or residence in inpatient rehabilitation programs. One hundred and fifty four regular IDU were eligible to participate in the New South Wales IDRS in 2003. Prior to commencing the interview, each participant was provided with information about the study as well as an assurance of confidentiality. Once the participant provided written consent for involvement in the study, a structured interview of approximately 45 minutes duration was conducted. No identifying data was collected at any time throughout the interview or recorded on the questionnaire. Responses were coded according to closed data fields on the interview schedule. IDU sampled for the IDRS are not intended to be representative of all IDU, but do provide important information about patterns of illicit drug use among IDU who are actively engaged in illicit drug markets, a group that we wished to examine in this study.

IDU who reported current engagement in sex work for money and or drugs are classified as sex workers for the purpose of this paper.

### Statistical Analyses

Differences in demographics and drug preference were analysed using chi square statistics. Differences in age of initiation into injecting drug use were analysed using the t test statistic. Mann Whitney tests were employed to analyse differences in drug use patterns (i.e. median days of use in the preceding six months) and expenditure on drugs.

### Limitations

The results of the current study should be interpreted as indicative of certain trends, given the relatively small number of sex workers sampled. Further research in Australia, examining issues raised in this study, needs to be conducted among larger groups of sex workers for more definitive results. Findings should also be interpreted within the context of street based sex workers, who differ from commercial sex workers in several domains [7,8,10]. While this limits the generalisability of findings to other sex workers, sampling street based sex workers serves the aims of this study well; many street based sex work markets function as an adjunct to illicit drug markets [12], with street based sex workers operating within close proximity to street based drug markets.

## Results

### Demographic Characteristics

The demographic characteristics of IDU according to sex work status are presented in Table 1. A total of 22 participants identified as having performed sex work for money and/or drugs in the month preceding interview. This represented 14% of the total IDU sample interviewed. This proportion was similar to those in previous years: 15% in 2002 and 7% in 2001 reported sex work as their main source of employment in the month preceding interview.

**Table 1:** Demographics of IDU by sex work status

	Sex workers % (n = 22)	Non-sex workers % (n = 132)
female	77	23
transgender	0	0
Age (M)	32	33
Years education (M)	8.9	9.7
ATSI	59**	28
not engaged in other employment	96	85
homeless	5	12
prison history	64	68
in drug treatment past 6 months	68	66
currently in drug treatment	45	47
criminal activity main income past month	0	28

**significant at p < 0.01

Among those in the 2003 sample who identified as engaging in sex work, 5 were male. The average age of sex workers (SW) was 32 years old (comparable to non sex workers (N-SW) who were, on average, 33 years old), and SW had completed an average of 8.9 years of education (compared to 9.7 years for N-SW). Sex workers were significantly more likely to identify as being of Aboriginal and/or Torres Strait Islander descent compared to N-SW (Table 1) (χ^2 ^= 6.94, df = 1, p < 0.01).

Ninety six percent of SW reported that they were not engaged in any other form of employment compared to 85% of N-SW. There were no significant differences between the two groups in the likelihood of reporting a prison history, participation in drug treatment or homelessness.

### Drug use history

The mean age of initiation into injecting drug use was significantly younger for SW (17.6 years) than N-SW (20.3 years) (t = 2.035, df = 152, p < 0.05). There were similarities between the groups with regard to the first drug they injected: heroin was the most common first drug injected, followed by methamphetamine. There was no difference in the mean number of drug classes SW and N-SW reported ever using (Table 2).

**Table 2:** Drug use history & current drug use of IDU by sex work status

	Sex workers % (n = 22)	Non-sex workers % (n = 132)
Age first injected (M)	17.6*	20.3
% heroin first injected	59	63
% amphetamines first injected	41	33
No. drug classes ever used (M)	10.6	10.1

% heroin drug of choice	96	83
% heroin injected most in past month	87	83
Daily or more injecting past month	82	65

Heroin		
injected last 6 months	100	96
Median days injected	175	170
Cocaine		
injected last 6 months	50	48
Median days injected	6	5
Methamphetamine (ice)		
injected last 6 months	36	35
Median days injected	36	5
Methamphetamine (speed)		
injected last 6 months	36	29
Median days injected	2	3.5
Benzodiazepines		
injected last 6 months	18	19
Median days injected	90	12
Alcohol		
used last 6 months	68	68
Median days used	24	18
No. drug classes used last 6 months (M)	7	6.5
Spent money on drugs yesterday	100*	77
Median amount spent on drugs yesterday	$145**	$100

*significant at p < 0.05

**significant at p < 0.01

### Current drug use

All but one of the SW reported heroin as their drug of choice (the remaining sex worker nominated benzodiazepines); among N-SW the majority reported heroin as their drug of choice, with smaller proportions nominating methamphetamine, cocaine, cannabis, and benzodiazepines. Heroin was reported as the drug most frequently injected in the month preceding interview among both SW and N-SW. Heroin was also most commonly reported as the last drug injected by both groups. There were no significant differences between proportions reporting injecting at least daily in the preceding month (Table 2).

Table 2 shows the classes of drugs used in the six months preceding interview and frequency of use during this period. Patterns of drug use among SW and N-SW were similar for most drugs with a few notable exceptions. Similar proportions reported using crystalline methamphetamine (ice) in the preceding six months, however SW reported using it on a median of 36 days compared with 5 days among N-SW. Likewise, although similar proportions reported intravenous benzodiazepine use in the preceding six months, SW reported a median of 90 days injecting compared to 12 days among N-SW. There was no difference in the mean number of drug classes SW and N-SW reported using in the preceding six months.

Sex workers were significantly more likely than N-SW to have spent money on drugs on the day preceding interview (χ^2 ^= 4.84, df = 1, p < 0.05), and to have spent significantly more on that day than N-SW (Table 2) (Mann-Whitney = 800, p < 0.01).

### Risk behaviours

Larger proportions of SW than N-SW reported borrowing used needles after someone else had already used them in the month preceding interview, and larger proportions had lent needles to others after they had used them. These differences were not significant. There was no difference in proportions sharing other injecting equipment in the preceding month. Likewise, there were no differences between proportions reporting last injecting in a public place, and usually injecting in a public place in the month preceding interview (Table 3).

**Table 3:** Self-reported risk behaviours & problems among IDU by sex work status

	Sex workers % (n = 22)	Non-sex workers % (n = 132)
borrowed needles in past month	14	5
lent needles in past month	23	11
last injected in public place in past month	36	26
usually injected in public place in past month	23	28
injection related problems past month	86*	55
attended mental health professional past 6 months	32	25
property crime in past month	32	31
drug dealing in past month	36	36
arrested in past 12 months	50	49

*significant at p < 0.05

Sex workers were significantly more likely to report injection related problems than N-SW (χ^2 ^= 6.32, df = 1, p < 0.05), with the most common injection related problems reported among SW being prominent scarring and bruising and difficulty injecting.

There were no differences between proportions of SW and N-SW reporting attending a mental health professional for mental health problems other than drug dependence in the preceding six months. Nor were there differences in proportions reporting engaging in property crime or drug dealing in the preceding month, or being arrested in the previous twelve months (Table 3).

## Discussion

This paper examined whether regular IDU who reported engaging in sex work were different from those who did not. Sex workers were more likely to identify as being of Aboriginal and/or Torres Strait Islander descent than non-sex workers, and this is consistent with previous research that has found that women who come from socially and economically disadvantaged ethnic minorities are over represented among sex workers [8,13,14], and also over represented among Australian injecting drug users [21,23-25]. These findings raise several implications for both health and drug treatment agencies, as well as for future research. Firstly, agencies providing health services for SW may need to consider tailoring programs to the needs of individuals who identify as ATSI, which could involve ATSI liaison personnel as part of outreach teams and services provided on site. Drug treatment programs also need to be more relevant for this population, as while ATSI are over represented among Australian IDU, they are under represented among IDU accessing drug treatment services [26]. There is general agreement among researchers that there is remarkably little published information available on ATSI IDU [27], and a paucity of research on what constitutes "culturally appropriate" treatment interventions for these populations [28]. Further research, establishing why so few ATSI IDU utilise available treatment programs, and identification of potential barriers for this group, is warranted in order to develop relevant programs that would encourage attendance.

There were no significant differences in drug types used by SW and N-SW, however SW initiated injecting drug use at a younger age. Again, these results are consistent with the literature that suggests that earlier age of initiation has been associated with a range of adverse outcomes later in life. Evidence suggests that those who have begun substance use by an early age are more likely to develop problematic substance use [29-32], engage in risky sexual behaviour [31,33], become involved in criminal activity [31], and complete fewer years of education [34]. Earlier initiates to substance use are also more likely to become more dependent, use for a longer time and have more drug-related problems. [35-40]. In the current study, among an already marginalised group of regular IDU, earlier initiation to drug use appeared to be associated with an additional risky behaviour – sex work. Research in Australia has illustrated these risks, with sex workers (particularly those who are street based) being more vulnerable to adverse contact with law enforcement, subject to physical assault, rape, kidnap, and being threatened with a weapon ([8,10,41,42]).

Earlier initiation of injecting drug use among this group indicates the need for greater emphasis on early intervention, in order to reduce the likelihood of young people entering sex work and/or developing problematic drug use [11]. For maximum effect, interventions should target several groups at different stages. Considerable research and public interest has been focused upon ways in which substance use among young people may be reduced, and to encourage those who have begun use at an early age to cease or moderate their use. Interventions have involved primary prevention (for example, drug education in schools or general population campaigns) ([43-45]); secondary interventions (such as targeted programs aimed at "at-risk" children) ([46]); and tertiary interventions (most often involving treatment for young persons who have developed problematic use, or interventions designed to reduce the initiation of injecting) ([47-50]).

Patterns of drug use were similar among both SW and N-SW, however results were indicative of heavier use of particular drugs among SW (i.e. ice and benzodiazepines). Due to small numbers of SW reporting recent benzodiazepine injection (n = 4) and ice use (n = 8), findings did not reach significance, and a larger sample size may highlight these trends more clearly. Trends of heavier drug use among SW are consistent with the literature documenting high levels of drug dependence in these groups [2]. The authors (AR and LD) are currently undertaking a study investigating a range of issues (including drug use patterns) among female street based sex workers and results should provide more definitive trends with regard to drug use in this group

Sex workers were more likely to have spent money, and to have spent more money on drugs on the previous day than N-SW, and this is most likely to be due to SW having more disposable income available to them than N-SW. However, it may also be an indicator of heavier drug use.

There were no differences between proportions of SW and N-SW reporting borrowing and lending used needles. Consistent with previous research [2,51], what seemed to be more indicative of the likelihood of borrowing needles was frequency of heroin use; 70% of IDU in the current study (regardless of sex work status) who reported borrowing needles were daily heroin users. People who had used the needle before them were reported as partners or close friends. Likewise, 74% of IDU (regardless of sex work status) who reported lending needles had used heroin on every third day or more (range 72–180 days) in the preceding six months (47% were daily users). These findings suggest that high levels of drug use may play a more important role in decisions to engage in risk taking behaviours than sex work does.

Sex workers were more likely to report injection related problems than N-SW however, given that the majority of SW were female, this finding may be more indicative of gender differences than sex work status *per se*. A paper describing the characteristics of clients attending the Medically Supervised Injecting Centre (MSIC) in Sydney [52] reported that females were twice as likely to report injection related problems than males, a finding that is consistent with other studies [1,8]. This finding is indicative of the need to ensure that these women have access to primary treatment services, and that there are no barriers to such services. Continued education campaigns, outlining strategies to minimise injection related harms also remain a priority.

Overall, these results suggest that the differences among injecting drug users who are sex workers and those who are not, are less striking than the similarities. Drug patterns were generally no different between these groups however, there was some indication of heavier use of crystalline methamphetamine and benzodiazepines among sex workers, and future research examining a larger sample is needed. Risk behaviours and poorer injection related outcomes appeared to be associated with factors other than sex work status (such as frequency of drug use and gender), perhaps suggesting that overall, injecting drug users who are sex workers may be at no greater risk of adverse outcomes (with the exception of the risks involved in street based sex work) than those who are not sex workers. It should be noted however, that this study did not examine condom use among injecting drug users, or the relationship between drug use and the safe outcome of sexual encounters, and these are undoubtedly issues of relevance for injecting drug users who engage in sex work.

## Conclusion

Few differences were found in the current sample of regular IDU who engaged in sex work compared with those who did not. There are however, several policy implications arising from differences that were apparent. Firstly, there needs to be an increased focus on more specific programs targeting SW who identify as ATSI, as well as further research into more culturally appropriate drug treatment services for this group. Second, greater emphasis needs to be placed on the continued development of primary, secondary and tertiary intervention programs targeting young people in a range of settings. There are some excellent programs currently available in Australia, and inclusion of more specific education regarding the risks involved in sex work as well as exploration of alternative employment opportunities would prove useful. Access to primary treatment settings for SW who are IDU is also important given the range of injection related problems they experience. Finally, due to relatively small numbers of IDU in this sample engaging in sex work, further research is required. In response, the National Drug and Alcohol Research Centre is currently undertaking research to assess a range of issues among female street based sex workers in Sydney. Education that targets safe sex practices among sex workers should remain a priority, given the high rate of problems encountered among this group, and the risks they face due to contact with multiple sex partners.

## Competing interests

The author(s) declare that they have no competing interests.

## Authors' contributions

AR was involved in collecting the data, performing statistical analysis, conducting a detailed literature review and drafting the manuscript. LD suggested the idea for the study and provided detailed structural comment on, and assistance with drafting the manuscript. CB was involved in collecting the data, providing detailed comment on the content, and assistance with drafting the manuscript. All authors read and approved the final manuscript.
